# Construction of ultrasonically treated collagen/silk fibroin composite scaffolds to induce cartilage regeneration

**DOI:** 10.1038/s41598-023-43397-z

**Published:** 2023-11-17

**Authors:** Shunan Yu, Xiong Shu, Lei Chen, Chao Wang, Xinyu Wang, Jinzhu Jing, Guoqiang Yan, Yanzhuo Zhang, Chengai Wu

**Affiliations:** 1 Department of Molecular Orthopedics, Beijing Research Institute of Traumatology and Orthopedics, Beijing, 100035 People’s Republic of China; 2 Animal Laboratory Laboratory, Beijing Research Institute of Traumatology and Orthopedics, Beijing, 100035 People’s Republic of China

**Keywords:** Biochemistry, Stem cells, Diseases

## Abstract

A novel tissue-specific functional tissue engineering scaffold for cartilage repair should have a three-dimensional structure, good biosafety and biological activity, and should be able to promote cartilage tissue regeneration. This study aimed to determine the effect of ultrasound-treated collagen/silk fibroin (Col/SF) composite scaffolds with good mechanical properties and high biological activity on cartilage repair. The characteristics of the scaffolds with different Col/SF ratios (7:3, 8:2, and 9:1) were determined by scanning electron microscopy, Fourier-transform infrared spectroscopy, and porosity, water absorption, and compression tests. In vitro evaluations revealed the biocompatibility of the Col/SF scaffolds. Results suggested that the optimal ratio of Col/SF composite scaffolds was 7:3. The Col/SF scaffolds induced adipose-derived stem cells to undergo chondrogenic differentiation under chondrogenic culture conditions. The efficiency of Col/SF scaffolds for cartilage regeneration applications was further evaluated using an in vivo model of full-thickness articular cartilage defects in New Zealand rabbits. The Col/SF scaffolds effectively promoted osteochondral regeneration as evidenced by macroscopic, histological, and immunohistochemical evaluation. The study demonstrates that ultrasound-treated Col/SF scaffolds show great potential for repairing cartilage defects.

## Introduction

Articular cartilage injury, which is one of the most common joint diseases worldwide, can result in pain, joint dysfunction, or disability^1–3^. Cartilage has limited self-repair capacity due to its avascular nature, low mitosis of chondrocytes, slow matrix turnover, and restricted supply of progenitor cells^4,5^. To date, microfracture, osteochondral transplantation, autologous/allogeneic chondrocyte transplantation, and other techniques and methods have been used clinically to treat articular cartilage defects, and have shown different degrees of efficacy^6,7^. However, there are still many problems to be solved, such as poor repair effect in a weight-bearing area, risk of relapse and limited donor area. Large cartilage defects are particularly difficult to treat and are associated with a series of problems, such as disease transmission and immune rejection, which restricts the progress of traditional therapy in the repair of osteochondral defects. Therefore, it is of great clinical significance to explore new and effective methods to promote cartilage regeneration and repair. Recent progress on tissue engineering holds great promise in regenerating cartilage by combining mesenchymal stem cells (MSCs) with biomaterials^8,9^, while the major challenge of inducing chondrogenic differentiation of MSCs is to minimize their de-differentiation^10,11^.

As a major component of the extracellular matrix (ECM), Col has good biocompatibility and promotes cellular adhesion, proliferation, chondro-differentiation, and biodegradability^12,13^. Col subjected to cyclic loading is rearranged and structurally destroyed, leading to a reduction in tissue toughness^14^. In previous studies, cartilage has been shown to respond to compressive loading by inhibiting swollen proteoglycan hydration through a complex collagen network^15^. The structural integrity of collagen networks thus plays an important role in cartilage regenerative scaffolds. However, the actual application of collagen is often limited by its poor mechanical properties, such as the inherently poor mechanical strength, and fast degradation.

The stress intensity, porosity, degradability, biocompatibility, proliferation, and differentiation of cells make SF one of the favorite scaffold materials for engineering chondroplasty^16–18^. SF is a regenerated protein derived from the silkworm, which maintains the advantages of silk fibers. At present, SF has widely been used in many biomedical fields, such as bone, cartilage, meniscus, ligaments, and intervertebral discs. Many studies have shown that SF can be processed into a variety of forms, including films, sponges, mats, gels, and scaffolds—all of which maintain excellent biocompatibility—and the degradation products are harmless to humans. However, silk proteins also have a lower affinity for cells, which can limit their use in scaffolds with 3D structures^19^. Considering both the advantages and disadvantages of these materials, a composite scaffold containing collagen and silk protein was developed and a porous scaffold was formed using the EDAC/NHS cross linker after ultrasound treatment of the two mixtures.

In addition, compared with other biomaterials, SF materials have controllable mechanical and degradation properties. By controlling the protein secondary structure between SF molecules, especially β-sheet, we can control the mechanical and degradation properties of the SF scaffolds.

**Figure 1:**
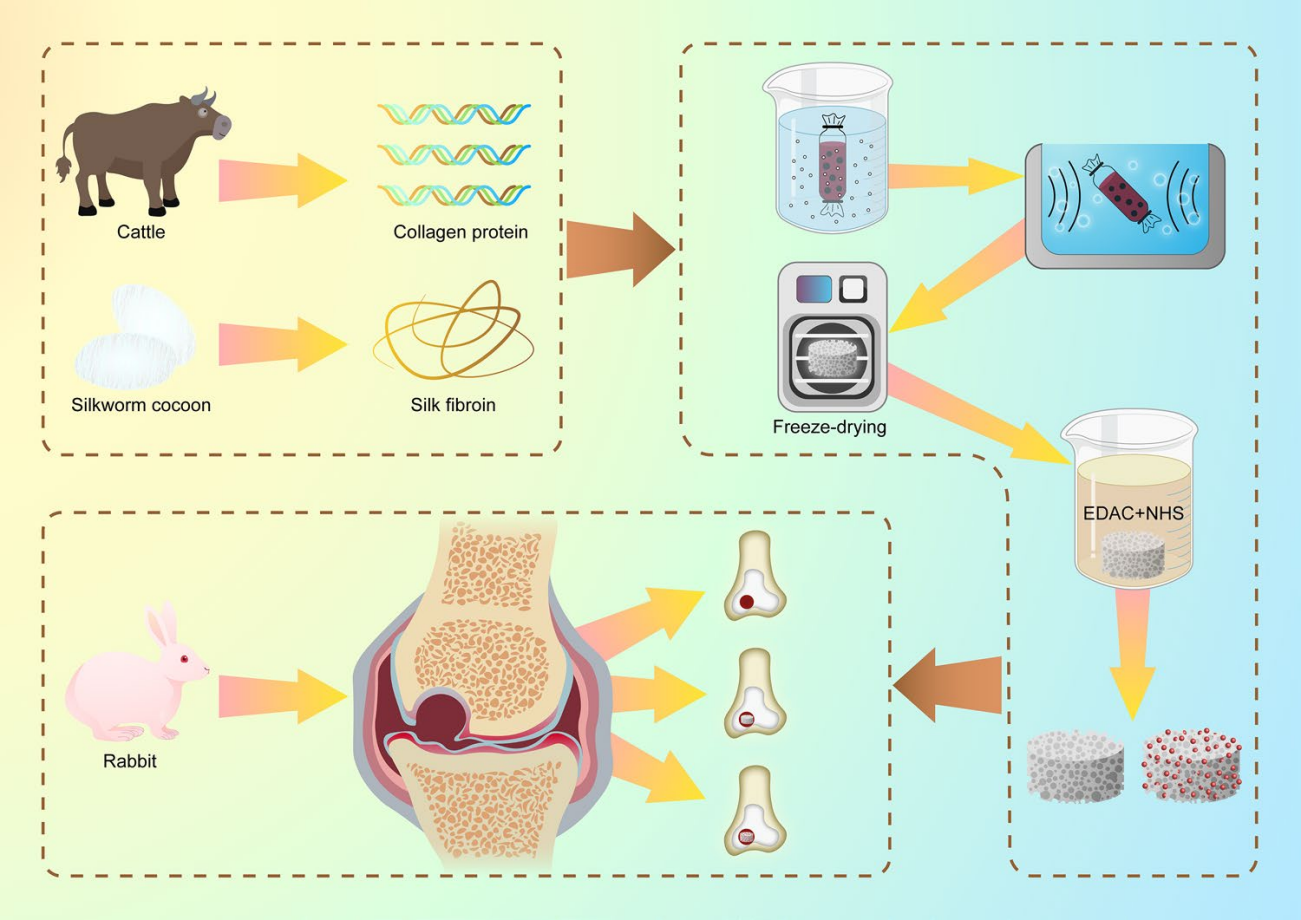
Fabrication of Col/SF scaffolds and their application for treating articular cartilage defects.

In this study, we fabricated a hybrid protein scaffold composed of Col and SF by sonication. It has been considered that the Col/SF scaffolds need reinforcement with complementary properties to gain optimal mechanical and biological functions. We prepared Col/SF scaffolds over a wide compositional range, and characterized their physical properties and biocompatibility in vitro that were appropriate for cartilage tissues. Furthermore, we observed the cell–Col/SF scaffold interactions, including cell aggregation, chondrogenic gene expression, sGAG deposition, and hydroxyproline (HYP). Based on the physicochemical and biological properties, the optimal ratio of the Col/SF composite scaffolds was 7:3. The cell–Col/SF scaffold constructs were implanted into cartilage defects in New Zealand rabbits (Fig. 1).

## Materials and methods

### SF purified solutions

Sericin was removed from the cocoon’s surface by placing the cocoon in a boiling NaHCO_3_ solution for 60 min. The degummed reconstituted silk fibers were rinsed with deionized water and then dried and sealed. The reconstituted silk fibers were then dissolved in tri solution (CaCl_2_·CH_3_·CH_2_·OH·H_2_O, molar ratio of 1:2:8 and silk protein fiber: tri solution volume ratio of 1:10) at 72 ± 2 °C with a magnetic stirrer for 1 h. The silk protein mixture was then placed in a dialysis bag, and dialysis was carried out in running tap water for 48 h before being replaced with deionized water once per hour for 48 h. After dialysis, the mass score was 3.5 wt% of the SF solution.

### Preparation of collagen

The fascia was first peeled from a bovine Achilles tendon, muscle and fat were removed, and the fascia was thoroughly ground using a slicer and tissue agitator. The protein was then agitated with 0.2 mol/L NaHCO_3_ for 6 h to remove any impurities. The Achilles tendon crumbs were then washed in distilled water to adjust the pH to 7.0, followed by whisking for 1 h with 75% ethanol to defat and then washing again in distilled water to return the pH to neutral. The cleaned crumbs of 1% bovine tendon were dissolved in a solution of acetic acid (pH = 3.5, 0.5 mol/L), and the appropriate amount of 750U/mg pepsin (50:1 bovine tendon crumbs to pepsin mass ratio) was then added to the solution at 4 °C and stirred for 60 h. After centrifugation, the mixture was centrifuged and the supernatant was collected at 1200 rpm. Collagen precipitation was collected by centrifugation of the supernatant at 1200 rpm after adjusting the supernatant’s pH to 5.5 with 10 mol/L NaOH. The collagen deposits were then dissolved in a 0.5 mol/L acetate solution, followed by washing with ultrapure water and centrifuging at 1200 rpm.

### Preparation of Col/SF composite scaffolds

The freshly prepared collagen and SF were mixed in a dialysis bag with a microelectric mixer attached to the handle according to the Col/SF mass ratios of 7:3, 8:2, and 9:1. The mixtures were then vibrated for 20 min using an ultrasound machine (100% vibrational power). After removing the bubbles, the mixtures were further concentrated with a sucrose solution (5 times its volume) for 6 h. The condensed material was then laid out and freeze-dried. Cross-linking of the lyophilized material was performed at 4 °C with 50 mM EDAC + 20 mM NHS dissolved in 95% ethanol for 24 h (0.9585 g EDAC and 0.23 g NHS dissolved in 100 mL of 95% ethanol). Following cross-linking, the Col/SF scaffolds were obtained by washing the scaffold three times with PBS and three times with deionized water.

### Fourier-transform infrared spectroscopy (ATR-FT-IR) and SEM

We undertook the exploration of the biochemical profile of Col/SF scaffolds using ATR-FT-IR. This was performed with a Thermo Scientific Nicolet iS20 Spectrometer (Thermo Scientific, Waltham, MA, USA), equipped with a Smart iTR ATR accessory with a diamond crystal. The ATR-FT-IR absorption spectra were obtained within the range of 4000–400 cm^−1^, utilzing 32 scans at a resolution of 4 cm^−1^. The scaffold morphology was characterized by scanning electron microscopy (SEM). Using a scalpel, the scaffold specimens were cut into cross-sectional and longitudinal sections. The scaffold structure was observed by SEM after gold–palladium coating of the sections.

### Porosity

The porosity was determined by a modified liquid displacement method. Anhydrous ethanol was added to the tube, and its volume was denoted V1. A dry scaffold sample was then added to the test tube and immersed in the solution for 5 min. Negative pressure was applied to degas the scaffold, at which point the volume of ethanol was recorded as V2. The scaffold sample was gently removed, and the remaining ethanol volume was recorded as V3. The mean values of three samples from each group were calculated.

The porosity of scaffolds was calculated using the following formula: porosity = (V1 − V3)/(V2 − V3) × 100%

$${\text{Porosity}}\, = \,\left( {{\text{V1}} - {\text{V3}}} \right)/\left( {{\text{V2}} - {\text{V3}}} \right) \times {1}00\% .$$

### Water absorption

Samples of three dried scaffolds were taken from each group and impregnated with 0.01 mol/L PBS (pH = 4) for 24 h to equilibrate. The weight of the scaffold after drying its surface was designated as M0, and after drying for 12 h in the dryer as M1.

The rate of water absorption was calculated as follows: (M0 − M1)/M1 × 100%

$$\left( {{\text{M}}0 \, - {\text{ M1}}} \right)/{\text{M1 }} \times { 1}00\% .$$

### Compressive testing

The scaffolds were first divided into three groups based on Col/SF ratios (7:3, 8:2, and 9:1), cut into cylinders of uniform size, and soaked in ultrapure water for 24 h before being placed in separate DMA Q800 instruments. For the stress–strain curve, the experimental module was set to DMA Strain Rate, the experimental method to Strain Ramp, the experimental camp to Compression, the experimental air bearing gas to Air, the experimental temperature to room temperature, and the experimental procedure as follows: Ramp strain − 30.000%/min to − 70.00%. The modulus of the sample was determined from the slope of the straight line fitted to each of the curves^20^.

### Cell culture

Human adipose-derived stem cells (hADSCs) were purchased from Procell Life Science & Technology Co., Ltd (Wuhan, China). The cells was cultured with Dulbecco’s Modified Eagle Medium/F12 (DMEM/F12) and 10% fetal bovine serum (FBS). All cultures were maintained in a 5% CO_2_ incubator at 37 °C. The medium was changed every 2 days.

### Cytotoxicity assay

Three sets of Col/SF scaffolds (7:3, 8:2, and 9:1) were soaked in 75% alcohol for 48 h and then rinsed five times with a sterile 0.9% saline solution. The scaffolds were then soaked in low-sugar DMEM medium (containing 10% fetal bovine serum) in a 37 °C water bath for 24 h at a rate of 1.25 cm^2^/mL. Following extraction, the leachate solution was filtered through a 0.22-μm membrane. L929 cells were divided into four groups and seeded into culture dishes at a concentration of 1 × 10^4^ cells/mL with 100 μL per well and six wells per group. After incubation for 24 h, the medium was removed, 200 μL DMEM was added to the control group, while 200 μL leachate solution was added to each of the three groups of scaffolds. On days 2, 4, and 7, a culture panel was prepared with 20 μL of 3-(4,5-dimethyl-2-thiazolyl)-2,5-diphenyl-2-H-tetrazolium bromide (MTT) (5 mg/mL) per well for a period of 4 h. Next, 150 μL dimethylsulfoxide (DMSO) had been added to the culture solution under complete vacuum and allowed to oscillate for 10 min before the absorbance was measured at 492 nm using a multiplex enzymatic marker.

To evaluate hADSCs adhesion, the scaffolds were first transferred to a 12-well plate. Each scaffold was then inoculated with 50 μL of cell suspension containing 5 × 10^5^ hADSCs and incubated in a humidified 5% CO_2_ incubator at 37 °C for 2 h. After 7 days of culture, hADSCs in the scaffold were assessed using the Calcein/PI Cell Activity and Cytotoxicity Assay kit (Beyotime, China) in line with the manufacturer's instructions. After 30 min of incubation with a live/dead staining solution, both live (green) and dead (red) cells were observed under a confocal microscope.

### Quantitative RT-PCR analysis

Gene expression of the cartilaginous markers *SOX9*, *COL2A1,* and *ACAN* was analyzed by quantitative PCR. Briefly, the harvested ADSCs/scaffold constructs (n = 3) were frozen with liquid nitrogen, and total RNA was extracted and purified using the Takara MiniBEST Universal RNA Extraction Kit (Takara, Japan) in line with the manufacturer's instructions following 7 or 14 days of incubation. RNA concentrations were then quantified using a NanoDrop One spectrophotometer, and cDNA was obtained using a PrimeScript II 1st Strand cDNA Synthesis Kit (Takara, Japan). Quantitative RT-PCR was then performed using the Applied Biosystems 7500 Fast Real Time PCR System (Bio-Rad) using a Bio-Rad PrimeScript RT reagent Kit with gDNA Eraser (Perfect Real Time). The primers used in this study are included in Table 1. The expression levels of genes were analyzed by the △△Ct method and normalized to the expression level of *GAPDH*.

**Table 1:** The sequence of primers.

Gene name		Sequence
GAPDH	F	CTCCCACTCTTCCACCTTCG
	R	TTGCTGTAGCCGTATTCATT
Col2a1	F	CACGCTCAAGTCCCTCAACA
	R	TCTATCCAGTAGTCACCGCTCT
Aggrecan	F	AGGTCGTGGTGAAAGGTGTTG
	R	GTAGGTTCTCACGCCAGGGA
Sox9	F	AGTACCCGCATCTGCACAAC
	R	ACGAAGGGTCTCTTCTCGCT

### Sulfated glycosaminoglycans (sGAG) and hydroxyproline (HYP) quantification

GAG and HYP levels were measured in accordance with previously reported methods^2^. The scaffold–cell complexes on days 7 and 14 were used for genetic and biochemical analyses (DS-DNA, GAG, and HYP levels). Quantification of DNA, GAG, and HYP was performed using SpectraMax Paradigm. After weighing with a microbalance, the scaffolds seeded with ADSCs (n = 3 in each group at each time point) were digested for 24 h in a pre-prepared papain solution (Sigma) at 60 °C overnight for the estimation of dsDNA content and GAG content. A total of 60 μL of the above-digested specimen reacted with a working solution of Hoechst 33,258 (2 μg/mL) in the dark at 37 °C for 1 h. The intensity was measured with an excitation wavelength of 360 nm and an emission wavelength of 460 nm. The readings were compared with the calf thymus DNA Standard Curve (Sigma). The total sulfated GAG content was assessed using dimethylmethylene blue assay (DMMB, Sigma). Briefly, 60 μL of the above-digested sample was mixed with DMMB reagent and allowed to react for 30 min at room temperature, and the absorbance was measured at 525 nm. The content of GAG was calculated relative to the standard curve obtained from the shark standard curve 6-sulfate (Sigma, USA). Collagen content was determined by quantifying the HYP content. Aliquots of the same digested solution were further hydrolyzed in HCl at 120 °C for 2 h, and HYP content was measured at 560 nm. HYP content was determined based on the HYP standard curve (Sigma). Normalization of GAG and HYP was performed using ds-DNA.

### Animal model

All animals used in this study were adult male New Zealand rabbits that weighed between 2 and 2.5 kg. The experimental animals were provided by Beijing Vital River Laboratory Animal Technology Company. All procedures of the animal experiments were approved by the Beijing Jishuitan Hospital Animal Care and Use Committee, Beijing, China, and all of the methods were performed in accordance with the institutional guidelines for care and use of animals. The study is reported in accordance with the ARRIVE guidelines (https://arriveguidelines.org). All efforts were made to minimize the number of animals sacrificed in the experiment and their discomfort. Breeding conditions were maintained with single-cage housing, free movement inside cage, temperature of 18–23 °C, relative humidity of 50–60%, 12-h light/dark cycle, and ad libitum access to chow. 18 New Zealand rabbits were randomly divided into three groups, namely, the control group, the Col/SF scaffold group, and the Col/SF-hADSC scaffold group. For surgery, the animals were anesthetized with pentobarbital sodium and were placed in the prone position once they lost the pain reflex response. The articular cartilage was dislocated after surgical incision, exposing the articular tissue. A cylindrical cartilage defect (4 mm in diameter and 2 mm in depth) was formed by drilling corneal rings. In the Col/SF-hADSC scaffold group, hADSC-seeded scaffolds were placed in the cartilage defect area. A Col/SF scaffold was inserted in the Col/SF scaffold group. After reduction of the patella, suturing layer by layer from the joint to the skin was performed. To prevent infection, the New Zealand rabbits were given penicillin intramuscularly. The animals were sacrificed three or six months after the surgery, and further studies were carried out.

### Macroscopic observations

The restored tissue was assessed in accordance with the International Society for Chondroplasty (ICRS) macroscore. Three different investigators blinded to the experimental groups graded tissue repair.

### Histology and immunohistochemistry

The tissue samples were fixed in 4% paraformaldehyde for 24 h, flushed with tap water for 12 h, and then decalcified using 12.5% EDTA for 8 weeks. For the decalcified samples, a gradient ethanol series was used for dehydration. Following paraffin embedding and sectioning, 5-μm-thick sagittal sections were stained using Safranin O and Fast Green solution, Toluidine Blue staining solution, Red Picrosirius solution, and antibody against collagen II (1:200, Ab34712, Abcam, USA) to visualize type II collagen in line with the previous protocol^21^. For antigen retrieval, the sample sections were digested with pepsin (Sigma Aldridge, USA) at 37 °C for 30 min.

### Statistical analysis

Statistical analyses were performed using the software GraphPad Prism 7.0. All experimental results were presented as the mean ± standard deviation. All the data were presented as mean ± standard deviation. Two-Way or One-way analysis of variance (ANOVA) test was used to compare the means among groups. While data from the same group were evaluated using Student's t-test. Significance levels were set to at *P < 0.05, **P < 0.01, ***P < 0.001.

### Ethics approval and consent to participate

All procedures of the animal experiments were approved by the Beijing Jishuitan Hospital Animal Care and Use Committee, Beijing, China and the study is reported in accordance with ARRIVE guidelines (https://arriveguidelines.org).

## Results

### FT-IR spectroscopy of the Col/SF scaffolds

**Figure 2:**
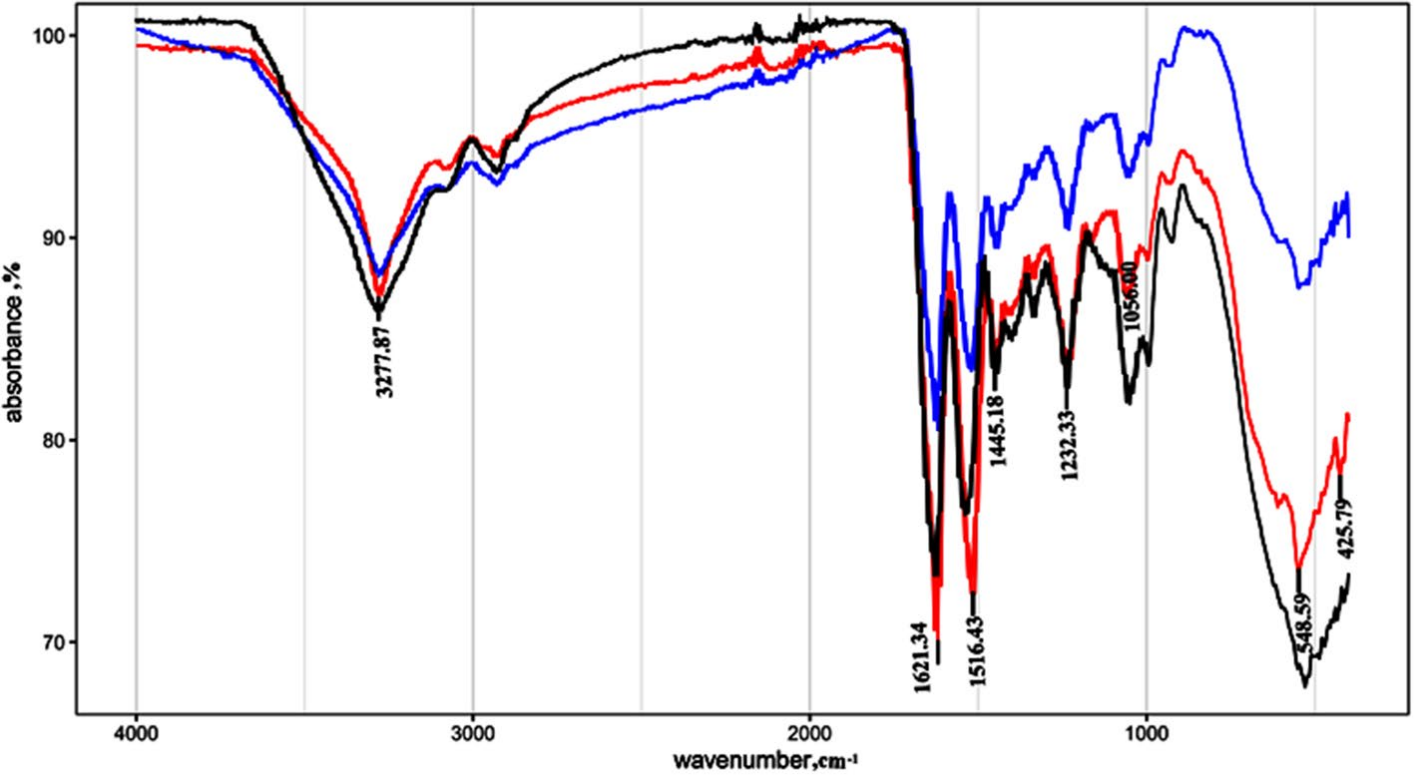
Fourier-transform infrared (FT-IR) spectroscopy was used to analyze and characterize the molecular structure of the scaffolds. The attachment ATR was placed in the spectrometer's optical path in a dry environment, and three scaffold samples were scanned first against the air background and then against the crystalline surface of the attachment ATR. Thirty-two scans and a range of test wavenumbers of 4000–400 cm^−1^ were then used to collect the sample in the infrared spectrum with a resolution of 4 cm^−1^. The red, blue, and black lines represent the Col/SF scaffolds at 7:3, 8:2, and 9:1 ratios, respectively.

FT-IR spectroscopy was used to analyze and characterize the molecular structure of the scaffolds (Fig. 2). The amino acid composition of Col and SF is dominated by glycine and alanine, and the characteristic peaks are hardly distinguishable. According to previous studies, the main spectral bands of Col and SF produced by the vibration of peptide bonds are the A, I, II, and III amides. Intake peaks in the amide I band at 1620–1637 cm^−1^ are dominant features of β-sheet structures, whereas random curling or α-helix results in broad and blurring peaks in amide II band. Figure 2 shows the ATR-FTIR spectrum of the Col/SF scaffold conformation transformation. Similar spectra of three scaffolds, namely, Col/SF (7:3), Col/SF (8:2), and Col/SF (9:1), showed the characteristic absorption peaks of amide I, amide II, and amide III of a β-sheet. As the proportion of collagen increased, the amide I zone moved from 1621 cm^−1^ to 1624 cm^−1^, and the characteristic peak intensity of β-sheet conformation decreased. It was usually accompanied by a conformational transition of filaments from the stable crystal structures of a β-sheet to unstable scroll structures. At the same time, IR uptake ratios from amide III to 1450 cm^−1^ (hereinafter AIII/A1450) are hallmarks of maintaining the collagen triple-helix integrity^22^. Table 2 shows a slight decrease in the collagen/proanthocyanin membrane ratio from 1.0 to a maximum of 0.99, indicating that proanthocyanin did not damage the triple-helix conformation, as the denatured collagen/gelatin ratio is 0.6. Hydrogen bonds, as opposed to water, are essential for stabilizing the native triple-helix structure at the molecular level. In this case, high concentrations of serin interacted with collagen to create new hydrogen bonds in the presence of ultrasound and cross-linkers. The triple-helix structure of the collagen has therefore been preserved. FT-IR analysis showed that the Col/SF (7:3) scaffolds were more stable, and the change in crystallinity provided excellent mechanical properties and resistance to degradation.

**Table 2:** The FT-IR absorbance ratios of the three Col/SF scaffolds at AIII/A1450 wavelength.

Col/SF	A_III_/A_1450_
7:3	0.99
8:2	0.87
9:1	0.85

### Characterization of the Col/SF scaffolds

**Figure 3:**
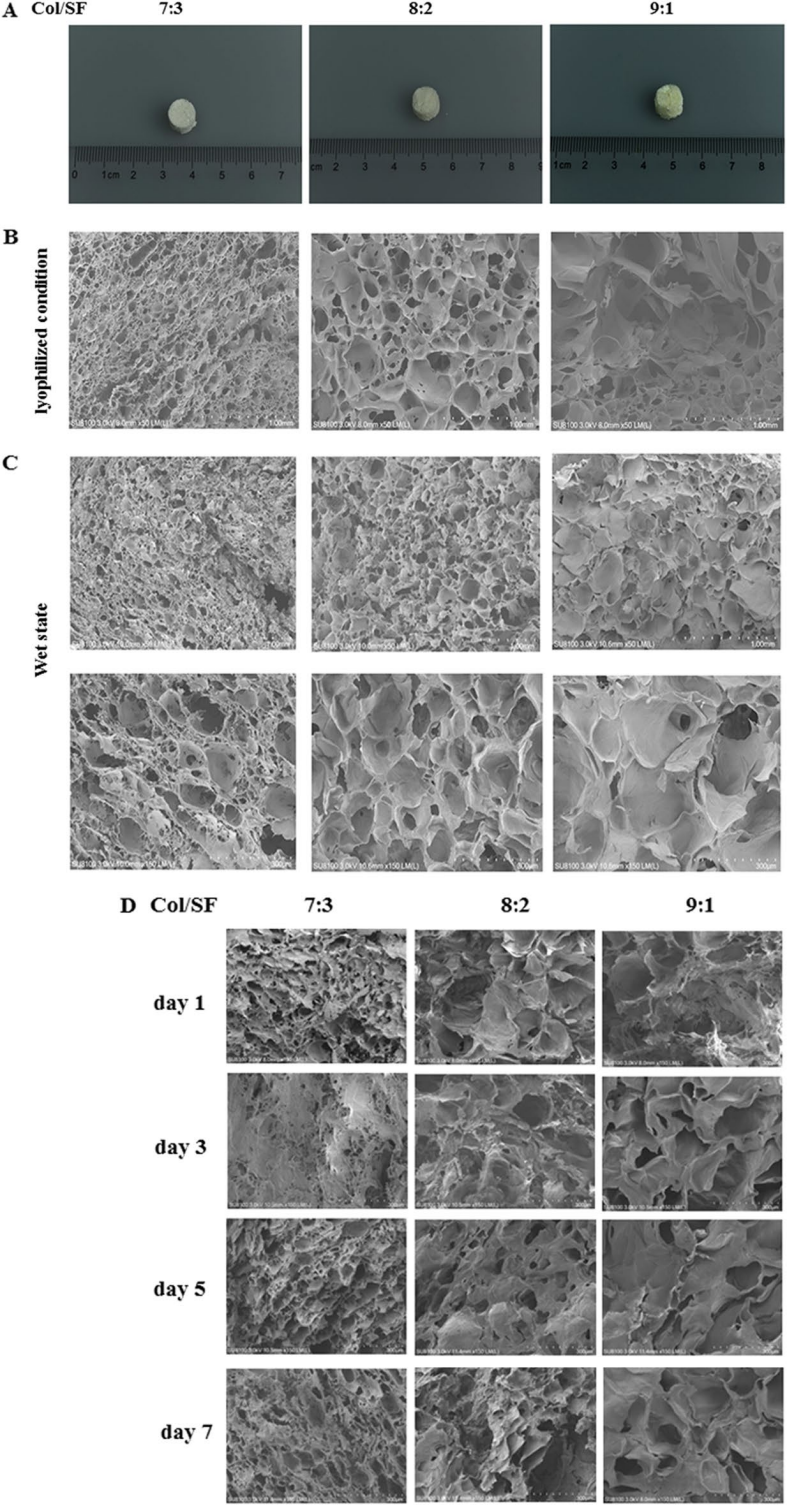

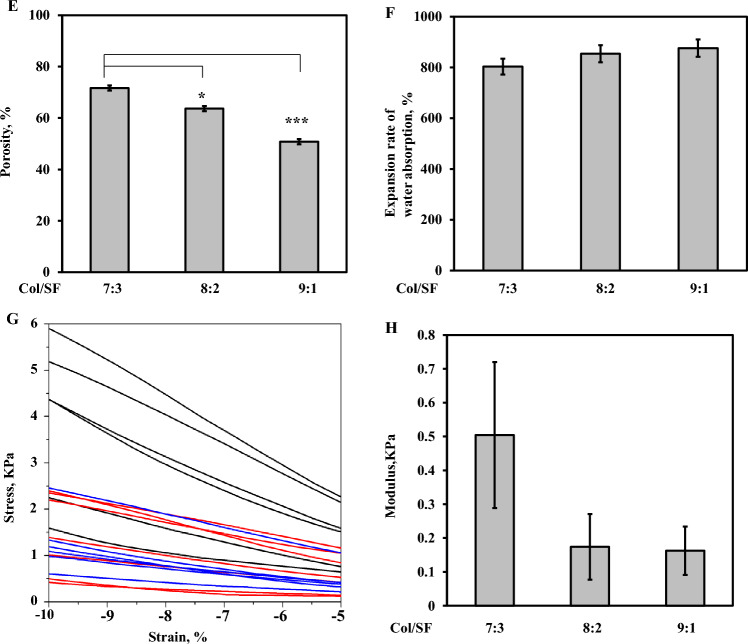
Characterization of the Col/SF scaffolds. (**A**) Gross appearance of Col/SF scaffolds of different ratios. (**B**) SEM images of the Col/SF scaffolds in a freeze-dried state with different Col/SF ratios (scale bar: 1.00 mm). (**C**) SEM images of the scaffolds with different Col/SF ratios after immersion in media for 24 h (scale bars, 1.00 mm (upper) and 300 μm (lower)). (**D**) SEM images taken after 1, 3, 5, and 7 days of L929 cell inoculation onto the scaffolds with different ratios of Col/SF (scale bar: 300 μm). (**E**) Water absorption of the scaffolds with different ratios of Col/SF. (**F**) Porosity of the scaffolds with different ratios of Col/SF. Stress–strain curves (**G**) and modulus (**H**) of the scaffolds with different Col/SF ratios. All data are shown as the mean ± SD of the experiments (n = 3).

A previous study has indicated that, compared with a single-protein gel^21^, Col/SF scaffolds could significantly enhance the physical, chemical, and biological properties. Col/SF scaffolds show excellent anti-deformation ability under different stress modes, and can maintain cell activity for a long time. To find the most suitable scaffold for cartilage regeneration, we prepared three kinds of mixtures with different proportions of SF and collagen. The filaments and collagen were mixed evenly in three proportions (Col/SF 7:3, Col/SF 8:2, Col/SF 9:1), ultrasonicated for 20 min, bubble-removed, concentrated with sucrose, and freeze-dried using EDAC/NHS cross-linking in 95% ethanol to create three scaffolds. SEM was used to assess the internal structure, cell adhesion, and growth of each of the three Col/SF scaffolds. The appearance of a Col/SF perforated scaffold pressed into a cylindrical shape is shown in Fig. 3A. SEM showed that different proportions of skeleton materials had differential internal structures in the lyophilized condition (Fig. 3B). The pore size of the scaffold is a major factor affecting cartilage regeneration^23,24^. The 7:3 Col/SF scaffolds exhibited a porous structure with an aperture of 150–250 μm. The Col/SF (7:3) scaffolds were also uniformly distributed with large spherical pores, which were connected by numerous small circular pores on the wall. The other two types of scaffolds presented with pores of larger aperture, poor connectivity, and poor smoothness (Fig. 3C). To further test the toxicity of the Col/SF scaffolds, L929 cells were spread over each of the three scaffolds, cultured for 1, 3, 5, and 7 days, and examined using SEM (Fig. 3D). On day 1, the cells appeared to strongly adhere to the scaffold Col/SF (7:3), while a lower number of cells adhered to the other two types of scaffolds. During the following days, the cells gradually proliferated and grew into the scaffold's inner pores. After 5–7 days, the cells in the Col/SF (7:3) scaffold were found to occupy most of the scaffold’s space. In contrast, the remaining space unoccupied by the cells was significantly larger in the other two scaffold types, in which the cells were attached to the scaffolds through multiple protrusions and were fixed to the pore walls. SEM revealed that the Col/SF (7:3) scaffold enhanced cell attachment and proliferation. In addition, the Col/SF (7:3) scaffold showed the highest porosity among the three scaffold types.

Based on the improved liquid displacement method for measuring porosity of scaffold materials, our results showed that the Col/SF (7:3) scaffold exhibited the highest porosity and best connectivity (Fig. 3E). The scaffolds with the different Col/SF ratios were placed in a PBS solution for 24 h, and their rates of water absorption expansion were measured (Fig. 3F). We found that the Col/SF ratio of 7:3 had the lowest rate of expansion of scaffolds water absorption and was more resistant to deformation. Compression experiments were also carried out on different proportions of scaffolds, and the stresses of the scaffolds with a ratio of 7:3 ranged from 1.5 to 6 kPa in the range of compression strain from − 5% to − 10%. The scaffolds (8:2 and 9:1) showed very similar results, with stresses ranging approximately from 0–1 kPa to 1–2.5 kPa (Fig. 3G) within the range of compressive strain from 5 to 10%. Moreover, Young’s modulus of the stent with the ratio of 7:3 was much higher than that of the other two types of scaffold (Fig. 3H). Together, we showed that the Col/SF (7:3) scaffold had better porosity, higher water absorption expansion rate, greater elastic modulus, and better cell proliferation, so it may be an ideal material for cartilage repair.

### Col/SF scaffold enhances chondrogenic differentiation of ADSCs in vitro

**Figure 4:**
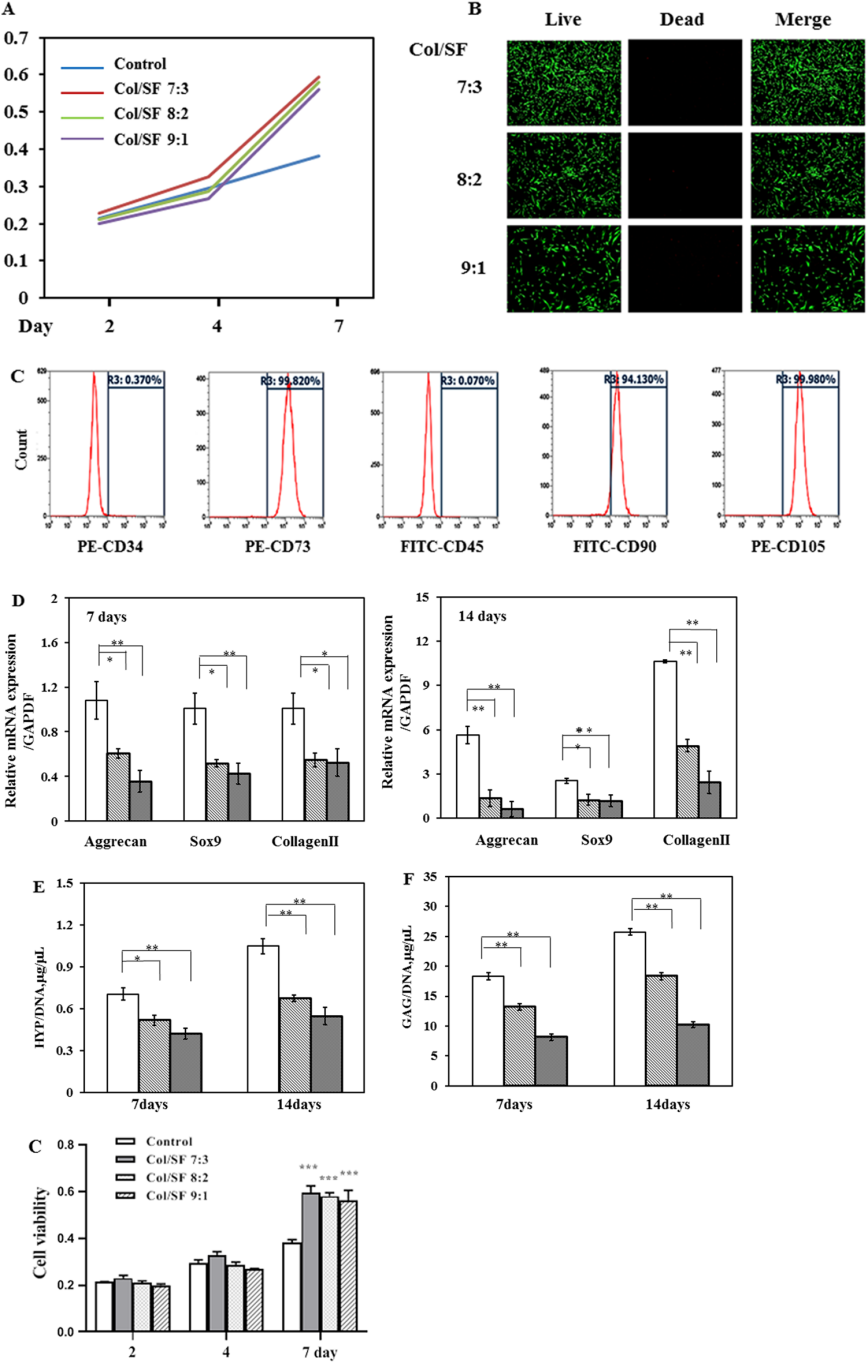
Col/SF scaffolds enhance chondrogenic differentiation of ADSCs in vitro. (**A**) L929 cell viability in scaffold extracts with different Col/SF ratios and control solution. (**B**) Live/dead assay of rabbit chondrocytes in scaffold immersed medium with different Col/SF ratios on fluorescence microscopy images. (**C**) Flow cytometry results of ADSC surface markers. (**D**) ADSCs grown on the Col/SF scaffold showed mRNA expression of cartilage-specific genes *acan, col2a1, and sox9* on days 7 and 14 based on Q-PCR. (**E**) The production of collagen in the scaffolds with different ratios of Col/SF was quantified using *HYP* assay. (**F**) Quantifying the production of cartilage matrix in the scaffolds with different Col/SF ratios via GAG assay. All data are shown as the mean ± SD of the experiments (n = 3). The white bar represents the Col/SF ratio of 7:3; the diagonal bar represents the Col/SF ratio of 8:2; and the black bar represents the Col/SF ratio of 9:1.

To evaluate the effect of the Col/SF scaffolds on cell proliferation, an MTT method was used to detect whether the scaffold was toxic to L929 cells. Figure 4A shows that the activity of the cells in the scaffold was similar to that in the control group during the first 4 days but increased significantly during days 4–7. A live/dead assay was used to detect the effect of scaffolds on the activity of rabbit articular chondrocytes (Fig. 4B). The live/dead assay revealed that there were few dead cells in view and that there was no significant difference between the scaffolds with the three different proportions. Our results indicated that Col/SF scaffolds were highly biocompatible with cell adhesion and proliferation. Overall, our study showed that the scaffolds with three Col/SF ratios had almost no cytotoxicity and little effect on articular chondrocyte activity.

To further investigate the effect of the scaffolds on the cartilage, we seeded ADSCs on these scaffolds and established a 3D culture system in vitro. Figure 4C shows the surface markers of ADSCs. We then measured the cartilage potential of ADSCs on these three scaffolds by analyzing their chondrogen-specific expression as well as the levels of HYP and GAG in the 3D in vitro culture system. GAG is one of the major components of the extracellular matrix of native cartilage and is the preferred detection index for assessing cartilage formation. HYP is a proline product catalyzed by the enzyme hydroxylase, and is an amino acid specific to collagen. HYP assays in cells may reflect changes in collagen metabolism. Compared with the scaffolds with ratios of 8:2 and 9:1, the scaffold with a ratio of 7:3 showed elevated expression of specific cartilage genes such as *ACAN*, *col2a1*, and *sox9* after 7 and 14 days of coculture (Fig. 4D). The 7:3 scaffold also showed higher expression than the 8:2 and 9:1 scaffolds, as measured by the levels of HYP and GAG (Fig. 4E,F). Taken together, the scaffold with the collagen to silk protein ratio of 7:3 presented better biocompatibility and significantly promoted differentiation of ADSCs into cartilage, so it warranted further in vivo investigations.

### Col/SF scaffold promotes cartilage defect repair in vivo

**Figure 5:**
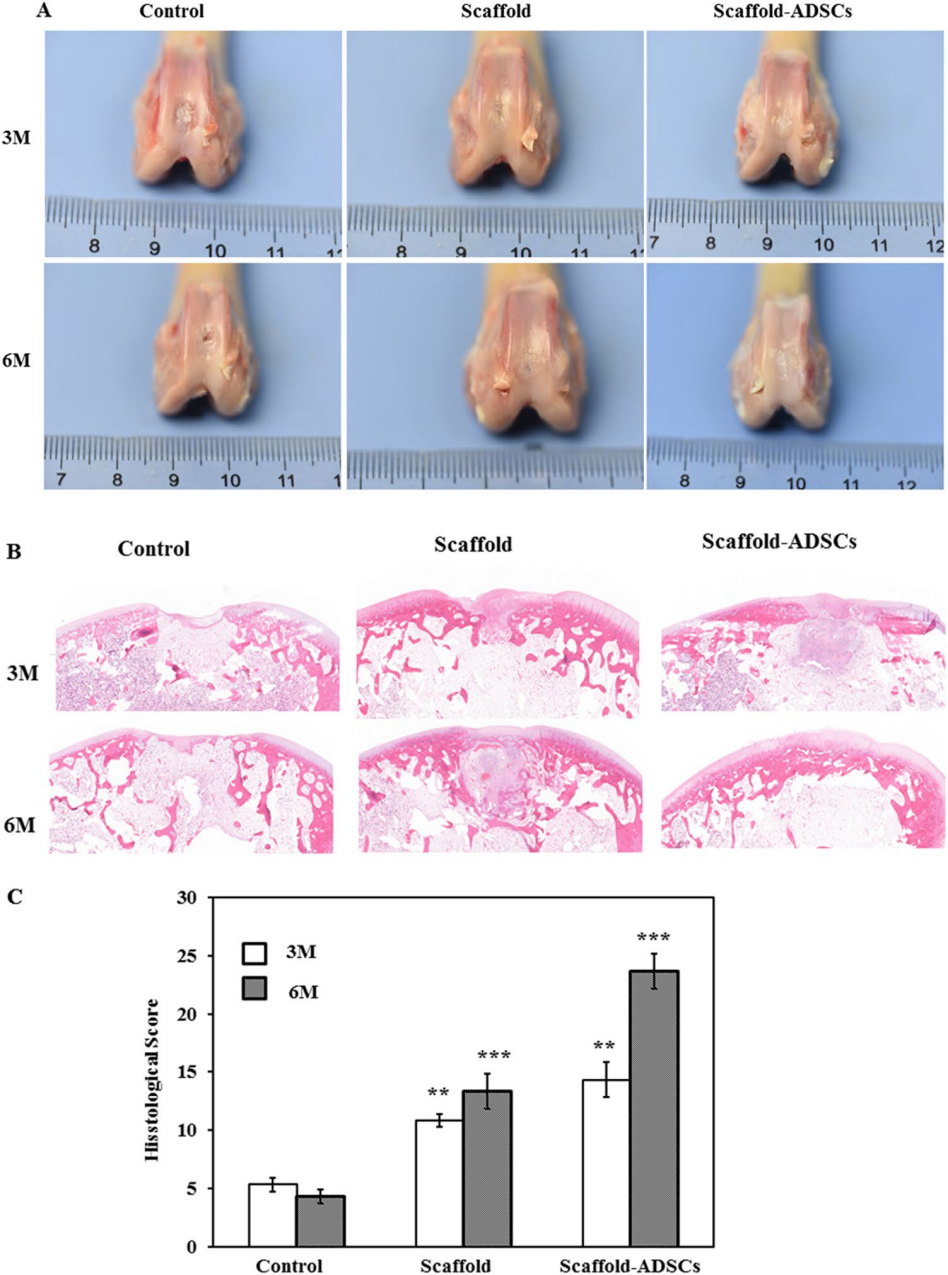

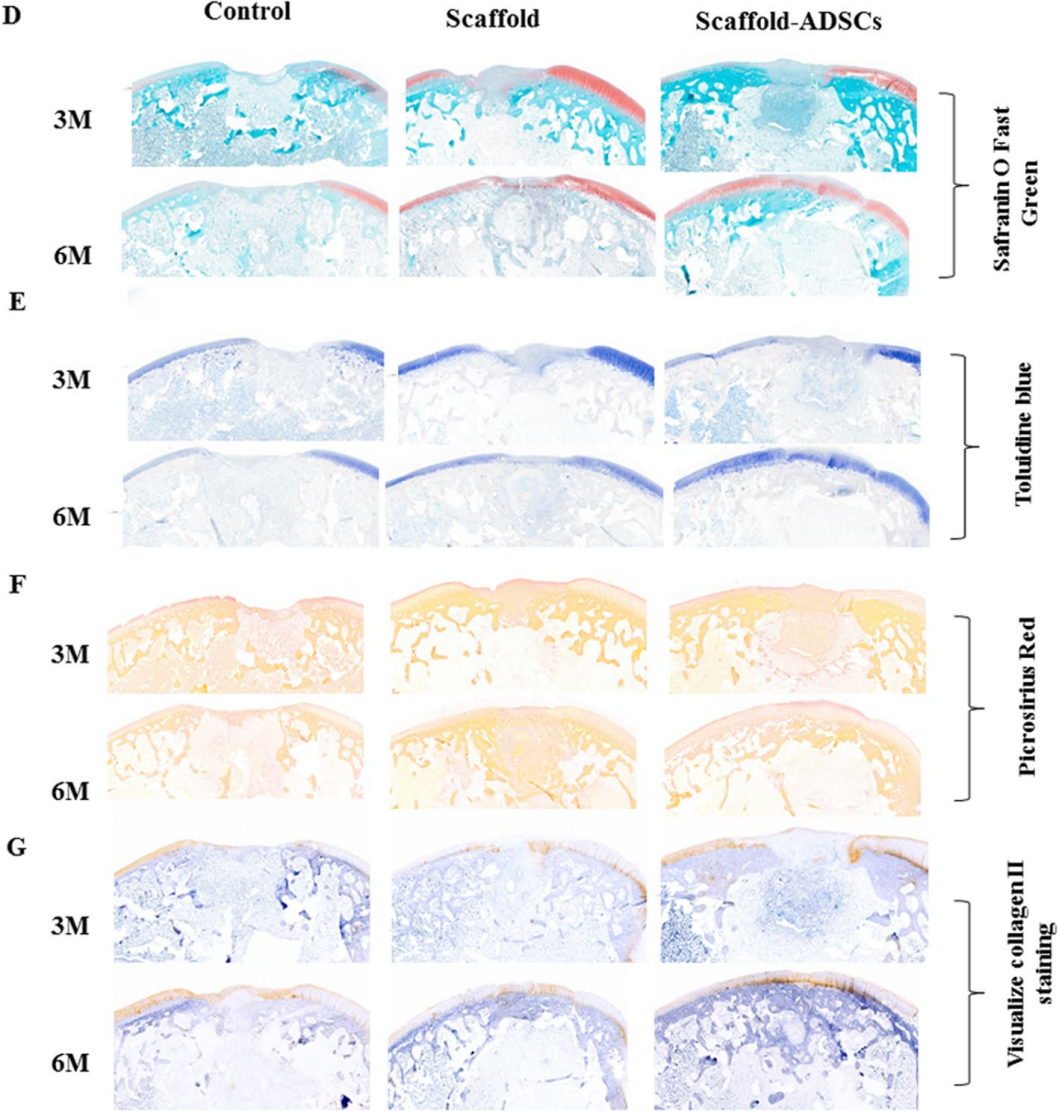
Col/SF scaffolds promote cartilage defect repair in vivo. (**A**) Macroscopic appearance of specimens taken at 3 and 6 months postoperatively. (**B**) Repaired cartilage was stained with H&E at 3 and 6 months. (**C**) Tissue score of repaired cartilage at 3 and 6 months. (**D**) Repaired cartilage was stained with Safranin O and Fast Green at 3 and 6 months. (**E**) Repaired cartilage was stained with Toluidine Blue at 3 and 6 months. (**F**) Repaired cartilage was stained with Picrosirius Red at 3 and 6 months. (**G**) Repaired cartilage was stained with immunohistochemistry at 3 and 6 months. All data are shown as the mean ± SD of the experiments (n = 3); scale bar, 500 μm.

To evaluate the ability of Col/SF scaffolds to promote cartilage regeneration in vivo, we established a cylindrical cartilage defect model in the center of the cochlear sulcus of the New Zealand rabbits and placed a Col/SF scaffold at the cartilage defect site^25^. In the Col/SF-ADSC scaffold group, almost the entire defect area was filled at 3 months postoperatively, showing a white surface that fused with surrounding tissue and partially repaired cartilage. In contrast, cartilage repair was deficient in the control and other groups, showing that the surface was rough, the surrounding tissue was not well fused, and the defect site was filled with a large amount of fibrous tissue (Fig. 5A,B). Cartilage repair using the Col/SF scaffolds was also assessed using the International Society for Chondroplasty (ICRS) score^7^. The ICRS score of the Col/SF-ADSC scaffold group was higher than that of the Col/SF scaffold group, and fivefold higher than that of the control group (Fig. 5C). Six months after the operation, the Col/SF-ADSC scaffold group showed significantly better performance, with the defect showing white pitching, smooth surface, and almost complete fusion with the surrounding tissue (Fig. 5A,B). Interestingly, compared with the control group, the Col/SF-ADSC scaffold group also showed some extent of cartilage repair, with a smooth surface area and considerable fusion with the surrounding tissue. The ICRS scores were significantly higher in the Col/SF-ADSC scaffold group than in the control group (Fig. 5C). We additionally performed histological and immunohistochemical staining using Safranin O and Fast Green, Toluidine Blue, and Picrosirius Red to visualize type II collagen in the femoral defect sites to further evaluate the effects of scaffolds on chondrocyte regeneration, extracellular matrix, and type II collagen synthesis (Fig. 5D-,lG). Three months after surgery, the defect areas in the control and the Col/SF scaffold groups were mostly filled with. In contrast, the Col/SF-ADSC scaffold group showed new cartilage and new bone tissue in the subcartilaginous bone. At 6 months after the surgery, the control group presented limited repair, while the Col/SF-ADSC scaffold group showed a higher number of regenerated chondrocytes, more abundant extracellular matrix, significantly restored collagen fiber network, and largely restored subchondral bone. Interestingly, in addition to partial cartilage regrowth in the Col/SF scaffold group, new bone tissue appeared in the subcartilaginous bone, suggesting that Col/SF scaffolds may have the ability to recruit stem cells. Taken together, these results demonstrate that the Col/SF-ADCSs scaffold is a bionic three-dimensional structure with excellent cartilage-repair ability.

## Discussion

The aim of this study was to investigate the effects of ultrasound treatment with the Col/SF scaffold in combination with ADSCs on cartilage regeneration. Properly functioning scaffold materials can provide a stable growth environment and differentiation space for the seed cells. In contrast to synthetic materials, Col is commonly used in the clinic as a key material for cartilage scaffolds, which as the first choice in cartilage repair is mainly due to good biocompatibility and extensive safety certification^20^. However, The Col scaffolds can lead to significant shrinkage, poor mechanical properties, and a limited ability to induce cartilage differentiation. SF are a kind of high-quality tissue engineering scaffolds with a low degradation rate and good mechanical properties^26^. Both Col/SF scaffold are considered to need reinforcement with complementary properties to gain optimal mechanical and biological functions.

In this study, we used bovine Achilles tendon to extract Col, which was predominantly type I Col. Type I Col is a heterogeneous molecule that consists of two α1 chains and one α2 chain, which support MSCs attachment, proliferation, and chondro-differentiation. However, type II Col–based stimulation of MSCs differentiation to chondrocytes is missing at the genetic level, as there is no significant difference in the expression of chondro-related mRNA over 21 days in the type II collagen gel (Sox9, COMP, COL1, COL2, COL10). Other studies have confirmed the upregulation of cartilage genetic markers at the protein level, but not at the gene level.

SF is a natural protein biomaterial extracted from silk, which has many advantages, such as controllable degradation, mechanical properties, excellent biocompatibility, and the ability to effectively maintain cell function^21–24^. Previous studies have shown that moderate ultrasound can promote the interaction between Col and SF. as shown in the present study, Col in combination with a high serin concentration after ultrasound also maintained a high mechanical performance, and in the right proportion resulted in a large β-Sheet that maintained scaffold stability and a stable triple helix structure that did not interfere with Col bioactivity and provided stronger support^25,27^. We prepare the ultrasonically treated SF/Col using EDAC/NHS over a wide compositional range (7:3, 8:2, 9:1), and observe the cell-scaffold interactions, including cell adhesion, spreading, and the survival of cells. We showed that the Col/SF (7:3) scaffold had suitable porosity, water absorption rate, and especially excellent elastic modulus, so it may be an ideal material for cartilage repair.

hADSCs have received more attention. Adipose tissue is abundant and easily obtain. It is important that hADSCs have immunomodulatory and anti-inflammatory properties. Therefore, hADSCs not only accelerates the time of induced cartilage formation in vitro, but also maintains engineered cartilage in vivo^28–30^. The results of Bordeaux et al. and Wang et al. support the use of xenogeneic ADSCs to promote articular cartilage repair^31,32^. We showed in vitro that the Col/SF scaffold significantly induced ADSCs to express the genes associated with chondrocytes. ADSC implantation in Col/SF scaffolds has been shown to increase cell adhesion and proliferation as well as mechanical properties due to the enhanced ECM secretion. ADSCs show greater potential for cartilage regeneration. As shown in our study, Col/SF scaffolds significantly increased the activity of ADSCs during cell proliferation.

In addition to internal structure, other physical aspects such as the stiffness and characteristics of the pores are also important for cell fate determination. It has been observed that adult stem cells sense the rigidity of the surrounding matrix and adjust their morphology and activity accordingly^33,34^. Hardness refers to the material's intrinsic resistance to strain and is an important characteristic of the environment that controls cellular activity. In general, anchorage-dependent cells exert contractile forces on the substrate and respond to the rigidity of the substrate by adjusting its adhesion force and the composition of the cytoskeleton. Subsequently, its regulation affects cell activities such as proliferation, differentiation, and secretion^35,36^. In general, scaffold stiffness is expressed as a function of Young's modulus and shear modulus^37^. Indeed, the studies have shown that ADSCs seeded on the soft matrix display lower expression of the smooth muscle marker (α-actin) and a significantly higher level of type II collagen compared to those seeded on the hard substrate^38^. Some studies have shown that MSCs grown in the soft sponge based on collagen (compression modulus 0.5 kPa) promoted upregulation of Sox9 (early chondro-genetic marker), whereas stiff sponges based on collagen (compression modulus 1–1.5 kPa) promoted the up-regulation of Runx2 (early bone-forming marker)^39^. In the present study, we found a mean Young's modulus of 0.5 kPa at a 7: 3 treated with ultrasound for the Col/SF scaffold and significantly increased early SOX9 expression in vivo.

In vivo experiments demonstrated that the cartilage repair effect can result from the Col/SF scaffolds with ADSCs that we studied. However, Col/SF scaffolds only produced disordered fibrous tissue with no hyaline cartilage. In contrast, Col/SF scaffolds loaded with ADSCs were completely replaced by the newly formed uniform cartilage tissue after implantation. This proves that through the Col/SF scaffold platform with ADSCs, the cartilage regeneration process is more effective. Therefore, compared with SF scaffolds, it is expected to significantly promote cartilage regeneration. The cartilage defect model and ADSCs derived from subchondral bone significantly improved cartilage regeneration.

However, the animal model in this study has certain limitations. To be precise, the cartilage defect site is not the main load-bearing site and cannot fully utilize the mechanical properties of the scaffold. Therefore, the cartilage defect model will need further optimization.

## Data Availability

All data in the current study are available from Cheng-Ai Wu author upon reasonable request.

## Abbreviations

ACAN: Aggrecan
ADSCs: Adipomesenchymal stem cells
ATR-FTIR: Attenuated total reflection Fourier transform infrared
Col/SF: Collage/silk fibrion
Col2: CollagenII
DMSO: Dimethylsulfoxide
MTT: 3-(4,5-Dimethyl-2-thiazolyl)-2,5-diphenyl-2-H-tetrazolium bromide
SEM: Scanning electron microscope
